# Supplementary material for: Globalizing manifold-based reduced models for equations and data
Source: Nat Commun. 2025 Jul 1;16:5722. doi: 10.1038/s41467-025-61252-9 (PMC12216585; doi:10.1038/s41467-025-61252-9)
Supplement: Supplementary file 1 — Supplementary Information [file 41467_2025_61252_MOESM1_ESM.pdf]

Globalizing Manifold-based Reduced Models for Equations and Data
Supplementary Information

Bálint Kaszás[1] and George Haller[1]

[1]Institute for Mechanical Systems, ETH Zürich, Leonhardstrasse 21, Zurich, 8092, Switzerland.

1 Further examples

1.1 The Euler example

We now recall an example from [1] and [2], which has originally been studied by Euler [3]. The system of equations reads as

$$\dot{x} = x^2 \tag{1}$$
$$\dot{y} = x - y. \tag{2}$$

The origin is a non-hyperbolic fixed point having a one-dimensional C^∞ center manifold. In fact, the whole phase space is foliated by one-dimensional center manifolds, and there is no distinguished one in terms of smoothness [2, 4]. Nevertheless, the center manifold can be parametrized as $y = h(x)$, which leads to the invariance equation

$$h(x)'x^2 = x - h(x), \tag{3}$$

which was studied by [3]. Seeking a power series approximation of $h(x)$ leads to

$$y = h(x) \sim \sum_{k=0}^{\infty} (-1)^k (k-1)! x^k \text{ as } x \to 0, \tag{4}$$

where the notation $\sim$ means that the asymptotic series need not converge for $x \neq 0$. The invariance equation (3) can be solved, for example, by multiplying by the

integrating factor $e^{-1/x}$, which leads to the expression

$$h(x,C) = Ce^{\frac{1}{x}} - e^{\frac{1}{x}}\mathrm{Ei}\left(-\frac{1}{x}\right) \tag{5}$$

where $C \in \mathbb{R}$ is arbitrary and

$$\mathrm{Ei}(z) = \int_{-\infty}^{x} \frac{e^t}{t} dt \tag{6}$$

denotes the exponential integral [5]. The center manifolds all have the coinciding asymptotic expansion (4), which has a zero radius of converge, as seen also in Fig. 1a.

Euler derived the differential equation (3) to assign a value to the sum of factorials with alternating signs, i.e., to the sum $1 - 2! + 3! - 4! + \ldots$. We refer to Chapter XII in [6] for historical context.

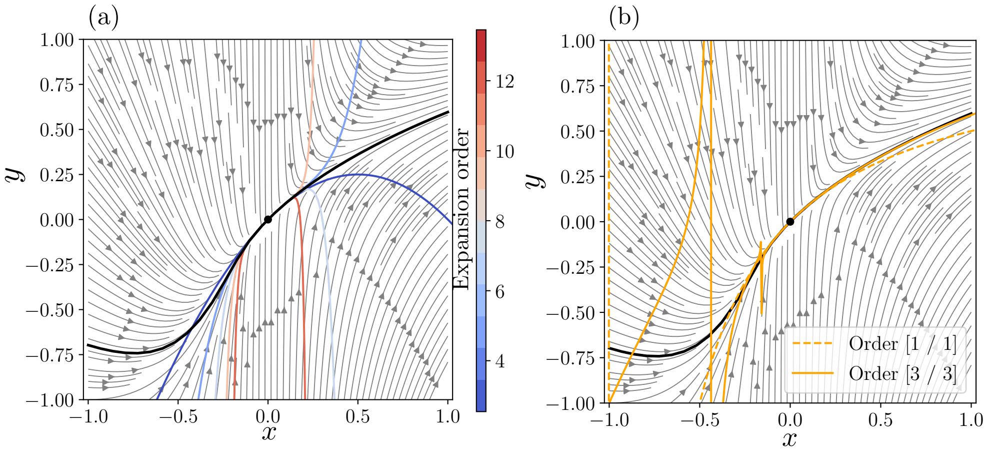

Supplementary Figure 1: (a): Phase portrait of (1) with the $C = 0$ member of the center manifold family (5) shown in black. (a): Evaluation of the Taylor expansion (4) up to order 15. (b): Padé approximants $[1/1](x)$ and $[3/3](x)$ of the series (4).

Computing Padé approximants of the series (4), however, reveals that even low-order approximants approximate the $C = 0$ member of the center manifold family for $x > 0$. Indeed, $h(x, 0)$ is an example of a Stiltjes function [7] and hence belongs to the rare class of functions for which the convergence of Padé approximants can be proven. Padé approximants of Stiltjes functions converge everywhere in the complex plane except along the negative real axis, where the original function $h(x, 0)$ has a branch cut.

This behavior is shown in Fig. 1b, where we see that even the low-order approximants show good agreement for $x > 0$. Their poles are concentrated on $x < 0$, mimicking the branch cut of the original function $h(x, 0)$. The approximants shown can be written as

$$y=[1/1](x)=\frac{x}{1+x} \quad y=[3/3](x)=\frac{x^3+8x^2+11x}{6x^3+18x^2+9x+1} \tag{7}$$

1.2 The Dauchot-Manneville model

The Dauchot-Manneville model is a planar model given by the system of equations

$$\begin{pmatrix}\dot{x}_1\\ \dot{x}_2\end{pmatrix}=\begin{pmatrix}s_1 & 1\\ 0 & s_2\end{pmatrix}\begin{pmatrix}x_1\\ x_2\end{pmatrix}+\begin{pmatrix}x_1x_2\\ -x_1^2\end{pmatrix}. \tag{8}$$

The model is bistable and shows strong non-normality at the trivial fixed point [8]. These properties are reminiscent of the subcritical transition to turbulence in shear flows [9]. For the parameter values $s_2<0, s_1<0$, this system has two coexisting stable fixed points, $p_1=(0,0)$ and p_3, and a saddle point, p_2. A one-dimensional manifold tangent to the slow spectral subspace connects the saddle to the two stable fixed points. We now compute the slow SSM of the origin, which is the same slow manifold observed by [8], hoping that this coincides with the connecting orbit. The nonresonance conditions of [2] are satisfied, and hence the slow SSM is analytic near the origin.

To find the parametrization of the slow SSM we assume that it can be written as a graph over the variable x_1. To construct this parametrization we assume a Taylor expansion of the form

$$x_2=W(x_1)=\sum_{n=1}^{\infty}w_nx_1^n \tag{9}$$

and find the coefficients w_n. Substituting (9) gives, to cubic order,

$$h(x_1)=-\frac{x_1^2}{2s_1-s_2}-\frac{2x_1^3}{(2s_1-s_2)^2(3s_1-s_2)}+O(x_1^4). \tag{10}$$

Fixing $s_1=-0.038$, in Fig. 2, we show the manifold computed up to increasing orders in the Taylor expansion, which is guaranteed to have a nonzero radius of convergence. To estimate the radius of convergence, [10] computed the accumulation point of zeros of the Taylor expansion (9) as in [11]. For orders $n<27$, all coefficients w_n are negative. At higher orders, $n\geq 27$, the sign of the coefficients alternates. Therefore, by the consequence of the Vivanti–Pringsheim theorem [12], the convergence limiting singularity is on the real axis at $x_1^s<0$, which is approximately given as $x_1^s=-0.01$. This limits the convergence for $x_1<x_1^s$, and therefore, the reduced-order model cannot contain any of the non-trivial fixed points.

The reduced dynamics on the SSM is obtained by substituting $x_2=h(x_1)$ into (8), which leads to

$$\dot{x}_1=s_1x_1+h(x_1)+x_1h(x_1). \tag{11}$$

The roots of (11) correspond to the predicted fixed points along the SSM. We show the reduced dynamics in Fig. 2b. As expected, outside the domain of convergence, the

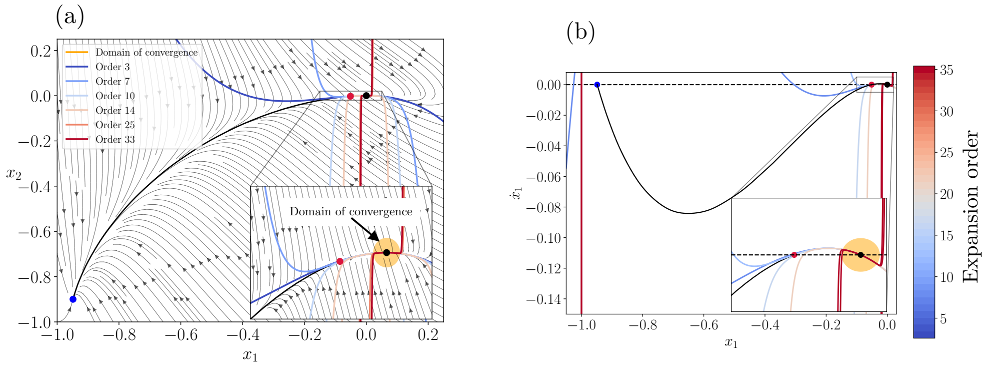

Supplementary Figure 2: (a) Phase portrait of the Dauchot-Manneville model (8) with $s_1 = -0.038$, $s_2 = -1$ and the Taylor series approximations of the slow SSM of the stable fixed point. The inset shows the domain on which the Taylor series converges. (b): The reduced dynamics on the SSM, obtained from the Taylor series approximation. The saddle is shown in red, the stable fixed points in blue, and black. The inset shows the same dynamics in the neighborhood of p_1 and p_2.

dynamics predicted from the Taylor expansion are very different from the true reduced dynamics computed numerically.

We now re-sum the divergent Taylor series outside its domain of convergence by computing its Padé approximants. Given the Taylor series representation of $h(x_1)$, the Padé approximants can be constructed by solving the linear equations presented in the main text. The sequence of the diagonal approximants, i.e. when $N = M$ generally has better convergence and approximation properties [7]. The diagonal Padé

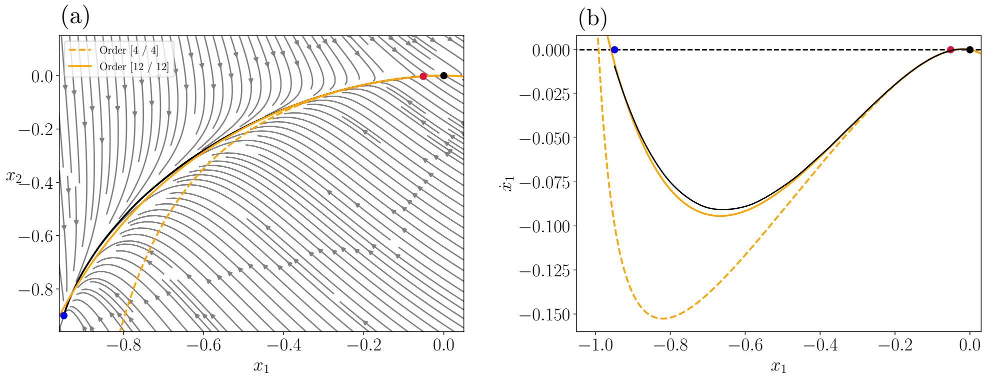

Supplementary Figure 3: Padé approximations of the slow SSM in the Dauchot-Manneville model with the same parameters as in Fig. 2. (a): the approximation in the phase space. (b): the reduced dynamics.

approximants of orders $M = N = 4$ and $M = N = 12$ are shown in Fig. 3. The

lower-order approximant can be written as

$$h(x_1) = \frac{-1.1x_1^2 + 40.55x_1^3 - 295.25x_1^4}{1 - 39.2x_1 + 347.58x_1^2 - 324.01x_1^3 - 647.28x_1^4}. \tag{12}$$

The approximation of the manifold is remarkably good over the entire region of interest spanning all three fixed points for a high-order approximant. Note that even the low-order approximant correctly predicts the three fixed points as well as their stability types, albeit with a non-negligible error in their location. Thus we successfully extended the local information contained in the Taylor expansion of the SSM to achieve a globally valid representation.

1.3 Invariant manifold with an imaginary singularity

It is often the case, that the convergence limiting singularity is not on the real axis, which means it is non-physical. As an example, consider the system

$$\dot{x} = x \tag{13}$$

$$\dot{y} = -y + \frac{2x}{(x^2+1)^2} \tag{14}$$

It can be verified that the unstable manifold of the origin is parametrized as

$$y = h(x) = \frac{x}{1+x^2} = \sum_{n=0}^{\infty} (-1)^{2n} x^{2n+1} \tag{15}$$

Note, however, that the first equality is valid for all $x \in \mathbb{R}$, the infinite sum representation is only valid for $|x| < 1$. Denoting the complex extension of the parametrization as $\hat{y} = h(\hat{x})$, it is apparent that the convergence of the series is limited by the two poles of $h(\hat{x})$ at $\hat{x} = \pm i$. Therefore, the radius of convergence is 1. There is no singularity along the real axis, and the parametrization remains well-defined for any $x \in \mathbb{R}$.

As expected, the Taylor series of $h(x)$ converges slowly, and only for $|x| < 1$. This is shown in Fig. 4. On the other hand, any Padé approximant $[N/M]$ with $N \geq 1$ and $M \geq 2$ is exact and can be evaluated for any $x \in \mathbb{R}$.

1.4 Shaw-Pierre oscillator

We recall the Shaw-Pierre system from [2], which is the forced and damped two-degree-of-freedom oscillator shown in Fig. 5a, whose equations of motion are given by

$$m\ddot{q}_1 + c(2\dot{q}_1 - \dot{q}_2) + k(2q_1 - q_2) + \gamma q_1^3 = \varepsilon \cos(\Omega t) \tag{16}$$

$$m\ddot{q}_2 + c(2\dot{q}_2 - \dot{q}_1) + k(2q_2 - q_1) = 0, \tag{17}$$

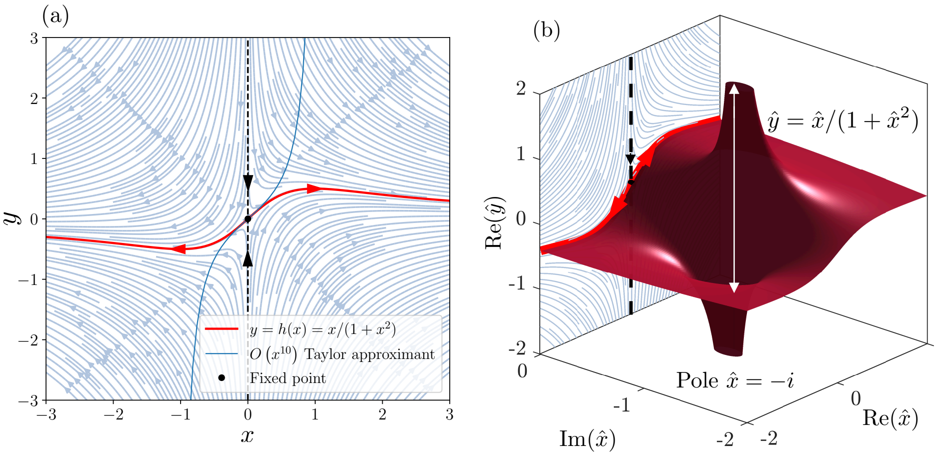

Supplementary Figure 4: (a): Phase portrait of (13) with the unstable manifold of the origin (red) and its order-10 Taylor approximant. (b): Visualization of the complex extension of the unstable manifold and the convergence limiting singularity at $\hat{x} = -i$.

with $m = 1$, $k = 3$, $c = 0.003$, $\gamma = 0.5$. The phase space is spanned by the four variables $(q_1, \dot{q}_1, q_2, \dot{q}_2)$. For $\varepsilon = 0$ the system has a stable fixed point at $(0, 0, 0, 0)$. For $\varepsilon > 0$ this fixed point perturbs into a periodic orbit, which represents the forced response of the system. Using SSMTool, we can calculate the two-dimensional SSM of the autonomous system ($\varepsilon = 0$).

To visualize the breakdown of the convergence of the Taylor series for high amplitudes $|p|$, we select an initial condition close to the origin of the unforced system at $p_0 = \bar{p}_0 = 10^{-3}$ and integrate the system backwards in time under both the reduced- and the full-order dynamics. The resulting trajectory and its Taylor approximation are shown in Fig. 5b and Fig. 5c. The Taylor approximation fails to describe an invariant manifold for $q_2 > 3$, which is also manifested by apparent self-intersections of the manifold.

The backbone curve, i. e. the function $\omega(\rho)$ is computed as

$$\omega(\rho) = 1.7320 + 0.0385\rho^2 - 0.0037\rho^4 + 0.0004\rho^6 + O(\rho^8), \tag{18}$$

and is shown in Fig. 6a. The plots show that the Taylor series approximation of the backbone curve converges for amplitudes up to $\rho \approx 3$. Moreover, the sign pattern of (18) shows alternating positive and negative coefficients, suggesting that the convergence limiting singularity is along the imaginary axis, similarly to the example in Section 1.3. This can be inferred from the calculations of [13], which we specialize to our case in Section 3.

Computing the forced response curves for increasing orders of the Taylor expansion, we find in Fig. 6b that they agree with the full-order computations as long as the response amplitude is smaller than $q_1 \approx 2$, as also inferred from the autonomous

analysis in Fig. 5. The full order computations were carried out using the numerical continuation package COCO [14].

Padé approximants, however have no problem approximating the true forced response even outside this domain of convergence. We compare the [3/3] and [5/5] approximants, which closely agree with each other. The forced response curves are shown in Fig. 6c. For example, the [5/5] approximant of the function $\omega(\rho)$ is given as

$$[5/5](\rho) = \frac{1.7320 + 0.3717\rho^2 + 0.0166\rho^4}{1 + 0.1924\rho^2 + 0.0074\rho^4} \tag{19}$$

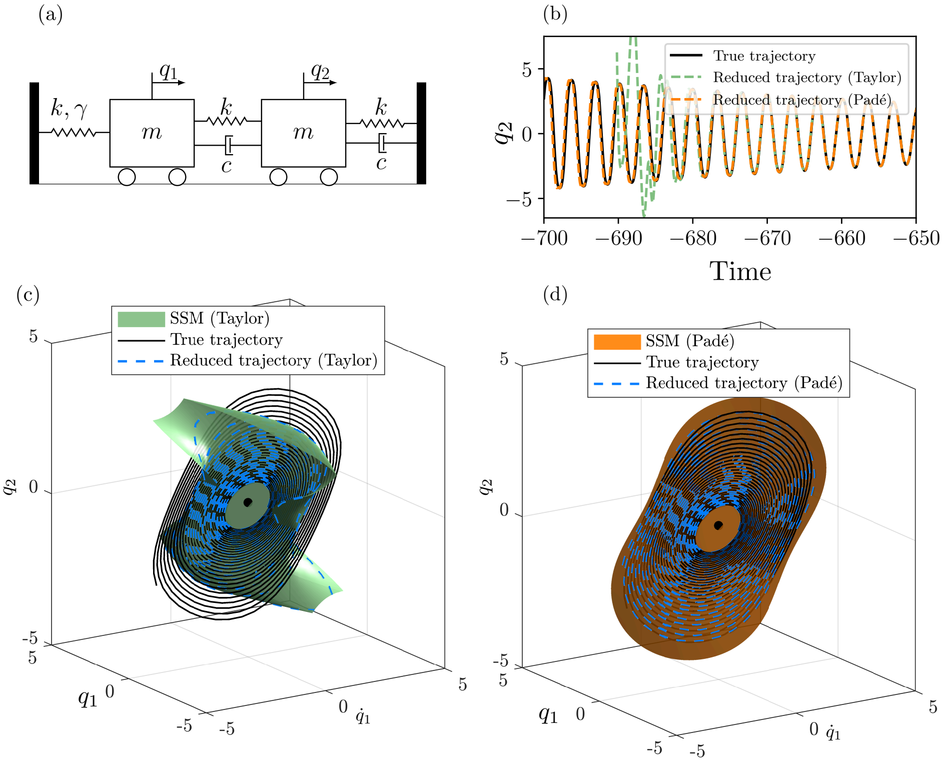

Supplementary Figure 5: (a): Schematic diagram of the Shaw-Pierre system. (b): Time series of a backward integrated trajectory started from $p_0 = \bar{p_0} = 10^{-3}$ in the full model (black), in an order-18 Taylor approximation (green) and the corresponding [5/5] Padé approximant (orange). (c): The trajectory in the phase space and the Taylor approximation of the SSM. (d): The Padé approximant of the SSM.

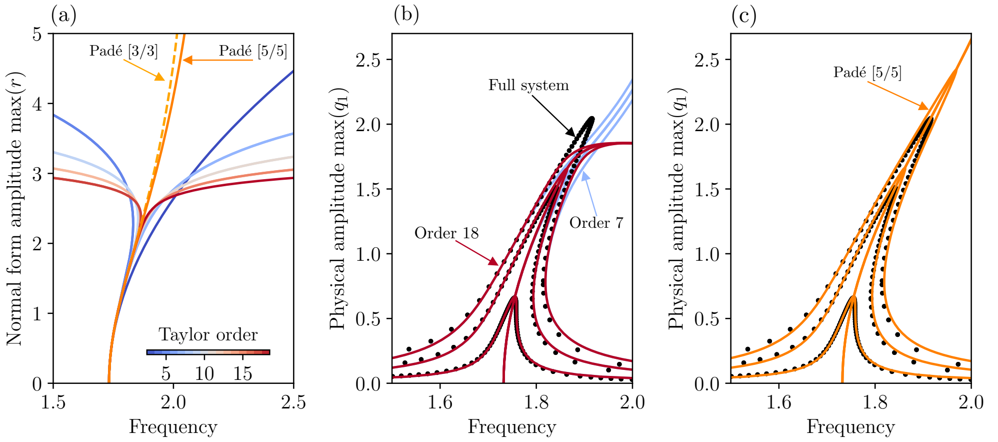

Supplementary Figure 6: Padé approximants of the backbone curves and forced response curves of the Shaw-Pierre oscillator. (a): Backbone curves expressed as $\omega(\rho)$. (b): Full-order response (black) with $\varepsilon = 0.05, 0.2, 0.3$ and its order-7 and 18 Taylor approximation. (c): Padé approximants of the response.

2 Kolmogorov flow

The Kolmogorov flow, in Fourier space, is governed by the following ordinary differential equations

$$\frac{d\hat{\omega}}{dt} = -\frac{1}{\mathrm{Re}}(k_x^2 + k_y^2)\hat{\omega} - \widehat{(\mathbf{u}\cdot\nabla)\omega} - 4\delta_{k_y,4}\delta_{k_x,0}, \tag{20}$$

for the discrete wave numbers $k_x, k_y = -12, ..., 12$, resulting in a total of 576 degrees of freedom. Based on [15, 16] we use a pseudo-spectral implementation. The nonlinearity is evaluated by introducing the stream function $\psi(x, y)$ as

$$\mathbf{u} = \begin{pmatrix} \partial_y \psi \\ -\partial_x \psi \end{pmatrix}. \tag{21}$$

The streamfunction can be recovered by solving the Poisson equation in Fourier space

$$(k_x^2 + k_y^2)\hat{\psi} = -\hat{\omega}. \tag{22}$$

The inverse Fourier transform of $\hat{\psi}$ then allows us to evaluate the nonlinearity $(\mathbf{u}\cdot\nabla)\omega$ in real space and take the Fourier transform subsequently. For dealiasing, we use the 3/2 scheme

3 Locating the convergence limiting singularity

The location of the convergence limiting singularity of a Taylor series expansion can be inferred from the sign pattern and the size of the Taylor coefficients. Here our main focus is to decide whether the singularity is on the real axis, as that would potentially indicate a genuine singularity of the function. This can be done by analyzing the sign pattern of the Taylor coefficients.

We specialize the analysis of [13] to the case of Taylor approximations of backbone curves and damping curves obtained from two-dimensional SSM-reduced models. The backbone curve $\omega(\rho)$ and the damping curve $\kappa(\rho)$ are even functions of their arguments. The sign pattern is governed by the closest singularity of the function in the complex plane. Consider the prototype even function with poles at $z = \pm re^{\pm i\theta}$, singularity given by

$$\begin{aligned} G(z) &= g(z) + g(-z), \text{ with} \\ g(z) &= \left(1 - \frac{z}{re^{i\theta}}\right)^{\nu} + \left(1 - \frac{z}{re^{-i\theta}}\right)^{\nu}. \end{aligned} \tag{23}$$

The Taylor expansion of $g(z)$ around $z = 0$ converges for $|z| < r$ and is given as [13]

$$g(z) = \sum_{n=0}^{\infty} 2(-1)^n \binom{\nu}{n} r^{-n} \cos(n\theta) z^n, \tag{24}$$

which allows us to write

$$G(z) = \sum_{n=0}^{\infty} 2 \binom{\nu}{2n} r^{-2n} \cos(2n\theta) z^{2n}. \tag{25}$$

The sign pattern of the Taylor coefficients is determined by the cosine term. In our examples, the signs of the Taylor coefficients of the backbone curve and the damping curve of the von Kármán beam, and the Shaw-Pierre oscillator showed an alternating sequence of positive, zero, and negative values. This indicates that $\theta = \pi/2$, i.e. the singularity is likely close to the imaginary axis.

4 Checking for singularities

Special care must be taken to avoid spurious singularities of Padé approximants, especially in the multivariate case. For the parametrization of the von Kármán beam, we chose the [5/4] Padé approximant. This was because the diagonal approximant [5/5] had a spurious curve of singularities in the neighborhood of the origin, as shown in Fig. 7b. Since no general pointwise convergence result is available for the Padé approximants, one must always check whether singularities are present in the approximants. We found that decreasing the order of the denominator by one was sufficient to eliminate this singularity, as shown in Fig. 7a. To verify the invariance of the manifold, we

compute a backward-trajectory started very close to the origin, as shown in the main text.

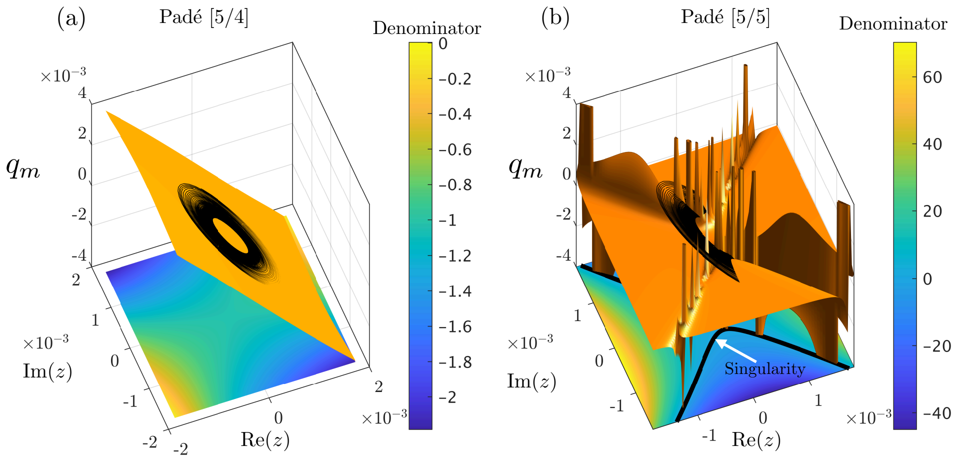

Supplementary Figure 7: Invariance of the Padé approximant of the parametrization. The orange surface is the Padé approximant corresponding to the tip of the beam. The value of the denominator is also shown, color-coded. (a): the [5/4] approximant is well-behaved near the origin and the backward trajectory (black). (b): The [5/5] approximant has a spurious singularity corresponding to the zero set of the denominator.

5 Properties of the chaotic von Kármán beam

We have shown that the gSSM model exhibits chaotic behavior when subjected to periodic forcing. We can further analyze this chaotic model. Specifically, we estimate the leading Lyapunov exponent of the chaotic attractor observed in the full model and the gSSM-reduced model. We perturb the initial conditions of the trajectories presented in the main text by a small perturbation of size 10^{-7} in the reduced coordinates. The rate at which the reference and perturbed trajectories deviate is governed by the leading Lyapunov exponent of the attractor [17].

Denoting the instantaneous distance between the perturbed and reference trajectories as $d(t)$, we expect that $d \sim e^{\lambda t}$ holds with $\lambda > 0$ on the attractors of both the full system and the gSSM-reduced system. Figure 8a shows the estimation of the leading Lyapunov exponent.

In addition, we also estimate the power spectral density (PSD) of the chaotic attractors. Based on long simulations of the reduced and full-order models lasting 200 forcing periods, we compute the frequency spectrum of the η_1 component of this time

series. The power contained in this spectrum is shown in Fig. 8b. The power spectrum computed on the gSSM closely matches the full-order simulation. The spectrum features a wide range of frequencies, indicative of chaotic behavior.

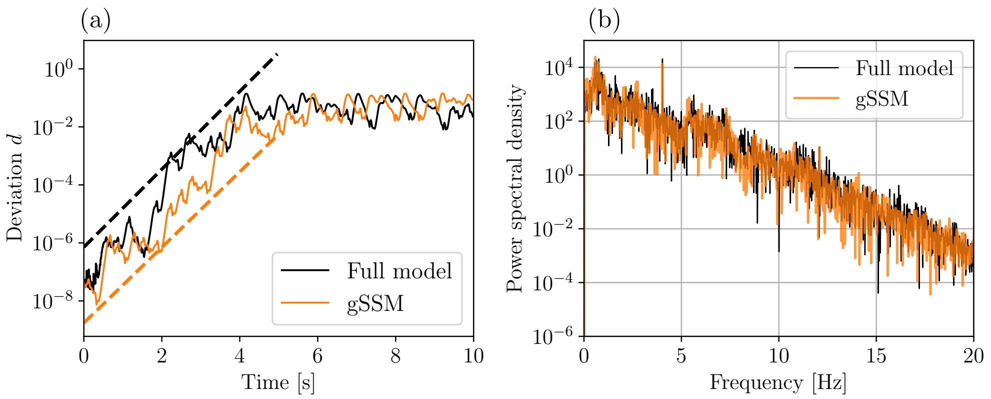

Supplementary Figure 8: (a): Estimation of the leading Lyapunov exponent based on the rate of divergence of nearby trajectories. Exponents are fitted to the initial exponential trend of the curves. We obtain $\lambda_{gSSM} = (3.0 \pm 0.02)\ 1/s$ and $\lambda_{full} = (3.1 \pm 0.05)\ 1/s$. The corresponding exponential functions are indicated with dashed lines. (b): Power spectral density of the attractors of the reduced and full-order models.

5.1 Nonautonomous model

The reduced dynamics on the SSM reads as

$$\mathbf{R}_\varepsilon(\mathbf{p}, \Phi) = \mathbf{R}(\mathbf{p}) + \varepsilon \sum_{|\mathbf{k}|=0}^{\hat{N}} \mathbf{S}_\mathbf{k}(\Phi)\mathbf{p}^\mathbf{k} + O(\varepsilon^2), \tag{26}$$

where $\hat{N} \geq 0$ is the approximation order for the forcing. In the main text, we have only considered the leading-order contribution with $\hat{N} = 0$, but the accuracy of the model can be improved by including higher-order terms as well. The corresponding gSSM-model can be constructed by computing an appropriate Padé-approximant for the forcing term in (26).

We compare predictions of the leading-order approximation presented in the main text and a [6/6] Padé-approximant computed with $\hat{N} = 17$ in Fig. 9. Qualitatively, the same type of chaotic dynamics is observed for the gSSM models, even when higher-order corrections are taken into account. However, for short times, the prediction error decreases even further when the nonautonomous terms are included. We also note that the polynomial SSM-model did not improve, even when we included the phase-dependent terms in (26). Specifically, the model experiences the same finite-time blowup as the leading-order approximation.

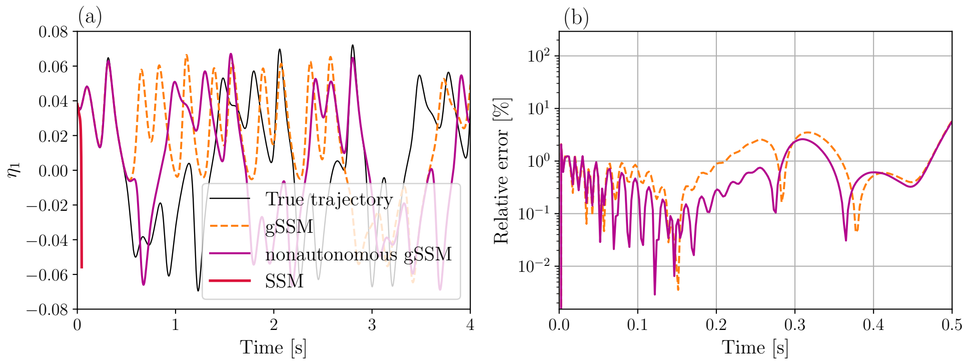

Supplementary Figure 9: (a): Time series of the reduced coordinate η_1 on a chaotic trajectory of the full system (black). Higher-order nonautonomous corrections are included in both the SSM-reduced forced model (red) and the gSSM-reduced model (violet). The leading-order approximation of the gSSM-reduced model is also shown orange. (b): Relative error of the reduced-model for short times.

6 Data-driven model of an inverted flag experiment

In the main text, we have shown that rational function regression is effective in modeling the reduced dynamics on a low-dimensional SSM. Denoting the reduced coordinates by $\boldsymbol{\eta} \in \mathbb{R}^2$, we approximate the reduced dynamics as

$$\dot{\boldsymbol{\eta}}(\boldsymbol{\eta}) \approx [N/M](\boldsymbol{\eta}) = \frac{\sum_{|\mathbf{k}|=0}^{N} \mathbf{a}_\mathbf{k} \boldsymbol{\eta}^\mathbf{k}}{\sum_{|\mathbf{k}|=0}^{M} b_\mathbf{k} \boldsymbol{\eta}^\mathbf{k}}. \tag{27}$$

In addition, we require that the denominator is non-zero at all points $\boldsymbol{\eta}_i$ in the training set.

We then determine the coefficients by minimizing the error

$$\mathcal{E}_r = \sum_{i=1}^{K} \left| \boldsymbol{\zeta}_i - \frac{\sum_{|\mathbf{k}|=0}^{N} \mathbf{a}_\mathbf{k} \boldsymbol{\eta}_i^\mathbf{k}}{\sum_{|\mathbf{k}|=0}^{M} b_\mathbf{k} \boldsymbol{\eta}_i^\mathbf{k}} \right|^2, \tag{28}$$

such that

$$\sum_{|\mathbf{k}|=0}^{M} b_\mathbf{k} \boldsymbol{\eta}_i^\mathbf{k} \geq \delta \quad \text{for } i = 1, ..., K, \tag{29}$$

for some small $\delta > 0$. We point out that without the regularization constraint (29), the regression can yield spurious singularities, which render the reduced model unusable in practice. In Fig. 10 we compare the vector fields obtained by polynomial regression (SSM) and rational function regression (gSSM). Due to singularities in the domain of interest, unconstrained rational function regression is unable to recover the correct phase portrait. The polynomial SSM-model and the constrained gSSM-models both capture the dynamical features of the reduced vector field accurately. However, outside

the range of the training data bounded by the stable limit cycle, the SSM-model starts to develop large gradients.

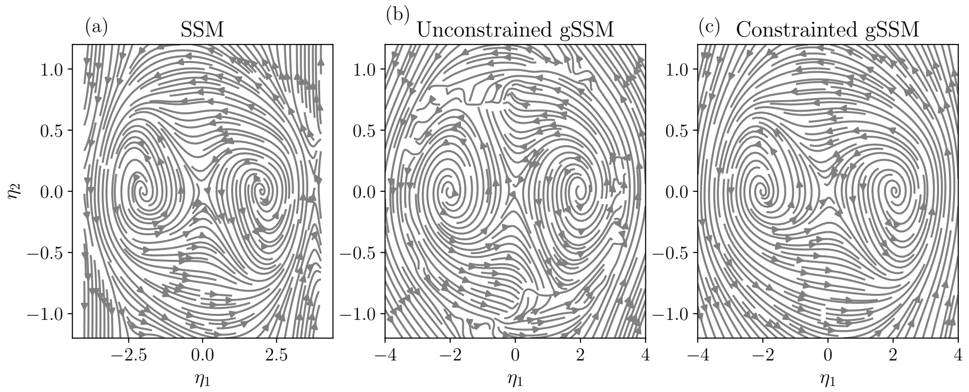

Supplementary Figure 10: Comparison of data-driven models of the inverted flag experiment. (a): Reduced vector field on the SSM approximated by an order-11 polynomial. (b): The vector field is approximated by a [5/5] rational function without the constraint (29). (c): Same as (b), but the constraint (29) is enforced.

Supplementary References

[1] Arnold, V.I.: Geometrical Methods in the Theory of Ordinary Differential Equations vol. 250. Springer, New York, NY (1988). https://doi.org/10.1007/978-1-4612-1037-5

[2] Haller, G., Ponsioen, S.: Nonlinear normal modes and spectral submanifolds: Existence, uniqueness and use in model reduction. Nonlinear Dynamics **86**(3), 1493–1534 (2016) https://doi.org/10.1007/s11071-016-2974-z

[3] Euler, L.: De seriebus divergentibus. Novi Commentarii academiae scientiarum Petropolitanae **5**, 205–237 (1760)

[4] Van Strien, S.J.: Center manifolds are not C^∞. Mathematische Zeitschrift **166**(2), 143–145 (1979) https://doi.org/10.1007/BF01214040

[5] Arfken, G.B., Weber, H.J.: Mathematical Methods for Physicists. Elsevier, Boston (2005)

[6] Bromwich, T.J.I.: An Introduction to the Theory of Infinite Series, 2nd edn. Macmillan and Company, London (1947)

[7] Bender, C.M., Orszag, S.A.: Advanced Mathematical Methods for Scientists and Engineers I. Springer, New York, NY (1999). https://doi.org/10.1007/978-1-4757-3069-2

[8] Dauchot, O., Manneville, P.: Local Versus Global Concepts in Hydrodynamic Stability Theory. Journal de Physique II **7**(2), 371–389 (1997) https://doi.org/10.1051/jp2:1997131

[9] Halcrow, J., Gibson, J.F., Cvitanović, P., Viswanath, D.: Heteroclinic connections in plane Couette flow. Journal of Fluid Mechanics **621**, 365–376 (2009) https://doi.org/10.1017/S0022112008005065

[10] Kaszás, B.: New Methods for Reduced-order Modeling and Uncertainty Quantification for Nonlinear Analysis of Fluid Flows. PhD thesis, ETH Zurich (2023). https://doi.org/10.3929/ETHZ-B-000648726

[11] Ponsioen, S., Jain, S., Haller, G.: Model reduction to spectral submanifolds and forced-response calculation in high-dimensional mechanical systems. Journal of Sound and Vibration **488**, 115640 (2020) https://doi.org/10.1016/j.jsv.2020.115640

[12] Remmert, R., Burckel, R.B., Remmert, R., Remmert, R.: Theory of Complex Functions vol. 122, 4. corr. print edn. Springer, New York Berlin Heidelberg (1998)

[13] Mercer, G.N., Roberts, A.J.: A Centre Manifold Description of Contaminant Dispersion in Channels with Varying Flow Properties. SIAM Journal on Applied Mathematics **50**(6), 1547–1565 (1990) https://doi.org/10.1137/0150091

[14] Dankowicz, H., Schilder, F.: Recipes for Continuation. Society for Industrial and Applied Mathematics, Philadelphia, PA (2013). https://doi.org/10.1137/1.9781611972573

[15] Chandler, G.J., Kerswell, R.R.: Invariant recurrent solutions embedded in a turbulent two-dimensional Kolmogorov flow. Journal of Fluid Mechanics **722**, 554–595 (2013) https://doi.org/10.1017/jfm.2013.122

[16] Farazmand, M., Saibaba, A.K.: Tensor-based flow reconstruction from optimally located sensor measurements. Journal of Fluid Mechanics **962**, 27 (2023) https://doi.org/10.1017/jfm.2023.269

[17] Ott, E.: Chaos in Dynamical Systems, 2nd edn. Cambridge University Press, Cambridge (2002)
