# Supplementary material for: Globalizing manifold-based reduced models for equations and data
Source: Nat Commun. 2025 Jul 1;16:5722. doi: 10.1038/s41467-025-61252-9 (PMC12216585; doi:10.1038/s41467-025-61252-9)
Supplement: Supplementary file 2 — Description of Additional Supplementary Files [file 41467_2025_61252_MOESM2_ESM.pdf]

Description of Additional Supplementary Files

Supplementary Movie 1: gSSM predictions of the oscillating beam.

Supplementary Movie 2: Comparing the SSM and gSSM predictions of the forced buckled beam.
